# Prognostic value of lipid metabolism‐related genes in head and neck squamous cell carcinoma

**DOI:** 10.1002/iid3.379

**Published:** 2020-12-05

**Authors:** Ying Xiong, Yu Si, Yisi Feng, Shipei Zhuo, Bozhen Cui, Zhigang Zhang

**Affiliations:** ^1^ Department of Otolaryngology Head and Neck Surgery, Sun Yat‐sen Memorial Hospital Sun Yat‐sen University Guangzhou Guangdong China; ^2^ Institute of Hearing and Speech‐Language Science Sun Yat‐sen University Guangzhou Guangdong China

**Keywords:** HNSCC, lipid metabolism, PI, TCGA

## Abstract

**Background:**

Altered lipid metabolism is involved in the development of many tumors. However, the role of dissimilar lipid metabolism in head and neck squamous cell carcinoma (HNSCC) is not fully established.

**Aims:**

Here, we sought to determine the prognostic value of lipid metabolism‐related genes in HNSCC.

**Methods:**

RNA‐seq data and clinical features of 545 HNSCC cases were obtained from The Cancer Genome Atlas database. A regulatory network of transcription factors‐lipid metabolism genes and a risk prognostic model of lipid metabolism‐related genes was developed using bioinformatics and Cox regression modeling. We used tumor immune estimation resource to analyze immune cell infiltration in patients with HNSCC based on the prognostic index (PI) of lipid metabolism‐related genes.

**Results:**

A total of 136 differentially expressed lipid metabolism genes were identified. Of these, 23 are related to prognosis. In addition to predicting HNSCC prognosis, 11 lipid metabolism‐related genes (ARSI, CYP27B1, CYP2D6, DGKG, DHCR7, LPIN1, PHYH, PIP5K1B, PLA2G2D, RDH16, and TRIB3) also affect HNSCC clinical features (stage, gender, and pathological stage). The PI of lipid metabolism‐related genes embodied the state of HNSCC tumor immune microenvironment.

## Graphical abstract

Lipid metabolism‐related genes effectively predicts head and neck squamous cell carcinoma (HNSCC) prognosis and reflects the state of immune cell in HNSCC.

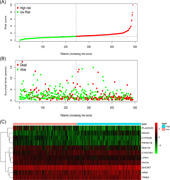

## 1 INTRODUCTION

Abnormal lipid metabolism is one of the most remarkable tumor metabolic defects. Increased lipid synthesis or absorption contributes to rapid cancer cell growth and tumorigenesis.^1^ Fabian et al.^2^ showed that factors involved in lipid metabolism can reduce cancer incidence. Recently, the autotaxin‐lysophosphatidic acid axis has been shown to promote pancreatic cancer progression by inducing interstitial cancer signal.^3^ In addition, lipids mediate intercellular communication in tumor immune microenvironment (TIME).^4^ Prostaglandin E_2_, produced by cyclooxy‐genase‐2, has been shown to promote TIME and block production of Type I interferon, preventing tumor elimination.^5^

Head and neck squamous cell carcinoma (HNSCC) is a multisite malignancy that affects about 600,000 patients annually, worldwide.^6^ Despite advances in treatment approaches, the 5‐year survival rate remains about 50%.^7^ In recent years, immunotherapy has gained a lot of interest for cancer treatment. However, it is only effective in 20%–30% of patients.^8^ Thus, it is necessary to study multicomponent antitumor responses and develop new anti HNSCC therapeutics. Current studies have confirmed that lipid metabolism promotes HNSCC invasion.^9^ However, other links between lipid metabolism and HNSCC are unclear.

Here, we sought to determine the survival role of lipid metabolism‐related genes and their therapeutic potential against HNSCC. We also systematically assessed the relationship between lipid metabolism‐related genes and overall HNSCC survival, as well as the correlation between prognostic index (PI) and six types of immune cells using bioinformatics. Findings from this study may improve personalized HNSCC treatment.

## 2 METHODS

### 2.1 Data collection and clinical specimens

RNA‐seq and clinical data on 501 HNSCC samples and 44 adjacent noncancer samples were downloaded from The Cancer Genome Atlas (https://cancergenome.nih.gov/). Four lipid metabolism‐related data sets (Reactome metabolism of lipids and lipoproteins, Reactome phospholipid metabolism, Hallmark fatty acid metabolism, and Kyoto Encyclopedia of Genes and Genomes (KEGG) glycerophospholipid metabolism) were obtained from the Molecular Signature Database v7.1 (MSigDB; https://www.gsea-msigdb.org/gsea/msigdb).^10^ Upon duplicates deletion, 856 lipid metabolism‐related genes remained. Next, 318 validated transcription factors (TFs) were obtained from Cistrome Cancer (http://cistrome.org/CistromeCancer/; *p* < .05). Immunohistochemistry (IHC) validation data were obtained from The Human Protein Atlas (HPA) database (https://www.proteinatlas.org/).

### 2.2 Bioinformatic analysis

Differentially expressed genes (DEGs) were analyzed using “limma” package on R V3.6.1 (https://www.r-project.org; log2 | fold change |>1, (FDR) < 0.05). Volcano plots and DEGs heatmap analyses were done using ggplot2 and pheatmap package, respectively.^11^ Database for annotation, visualization and integrated discovery v6.8 (https://david . ncifcrf. gov), was used for gene ontology (GO) and KEGG enrichment analyses. Ggplot2 package was used to visualize GO term and KEGG enrichment results. Mutation analyses were done on CBioportal (https://www.cbioportal.org/).^12^ The TFs‐lipid metabolism‐related genes regulatory network was mapped using Cytoscape v 3.7.0 (cor = .4, *p* < .001).^13^

### 2.3 Prognostic analysis

The R survival package was used to assess relationships between lipid metabolism‐related genes and overall survival (*p* < .05) and to plot survival curves. Prognosis related lipid metabolism genes were visualized on forest maps. Multivariate Cox analysis was used to select signature genes and determine the PI for each patient (*p* < .05). Risk curves were drawn using the pheatmap package. ROC maps were created using the survival ROC package. Univariate and multivariate independent prognostic analysis by the survival package were used to determine if PI could be used as an independent prognostic indicator.

### 2.4 Correlation analyses

Correlation maps between clinical features and signature genes were drawn using beeswarm package. Tumor infiltration immune cells data were obtained from tumor immune estimation resource (TIMER; https://cistrome.shinyapps.io/timer/) and included data on B‐cells, CD4^+^ T‐cells, CD8^+^ T‐cells, neutrophils, macrophages, and dendritic cells.^14^ These raw data were used to construct a relationship landscape between PI and six immune cell types (*p* < .05).

### 2.5 Statistical analysis

R software was used for results visualization. Differences between two groups were compared using independent *t* test. *p* < .05 indicated statistically significant differences.

## 3 RESULTS

### 3.1 Summary of results

Our analysis identified 4783 DEGs. Of these, 136 are associated with lipid metabolism and 63 are TFs. Differentially expressed lipid metabolism‐related genes were subjected to GO term and KEGG pathway analysis. We then assessed the mutations of the prognosis‐related lipid metabolism genes and constructed a regulatory network of TFs and lipid metabolism‐related genes. We further established a prognosis model of lipid metabolism‐related genes and divided all patients into two groups based on PI. The gene set was validated on HPA database and their relationship to clinical traits and immune cells determined.

### 3.2 Identification of differentially expressed lipid metabolism‐related genes

**Figure 1:**
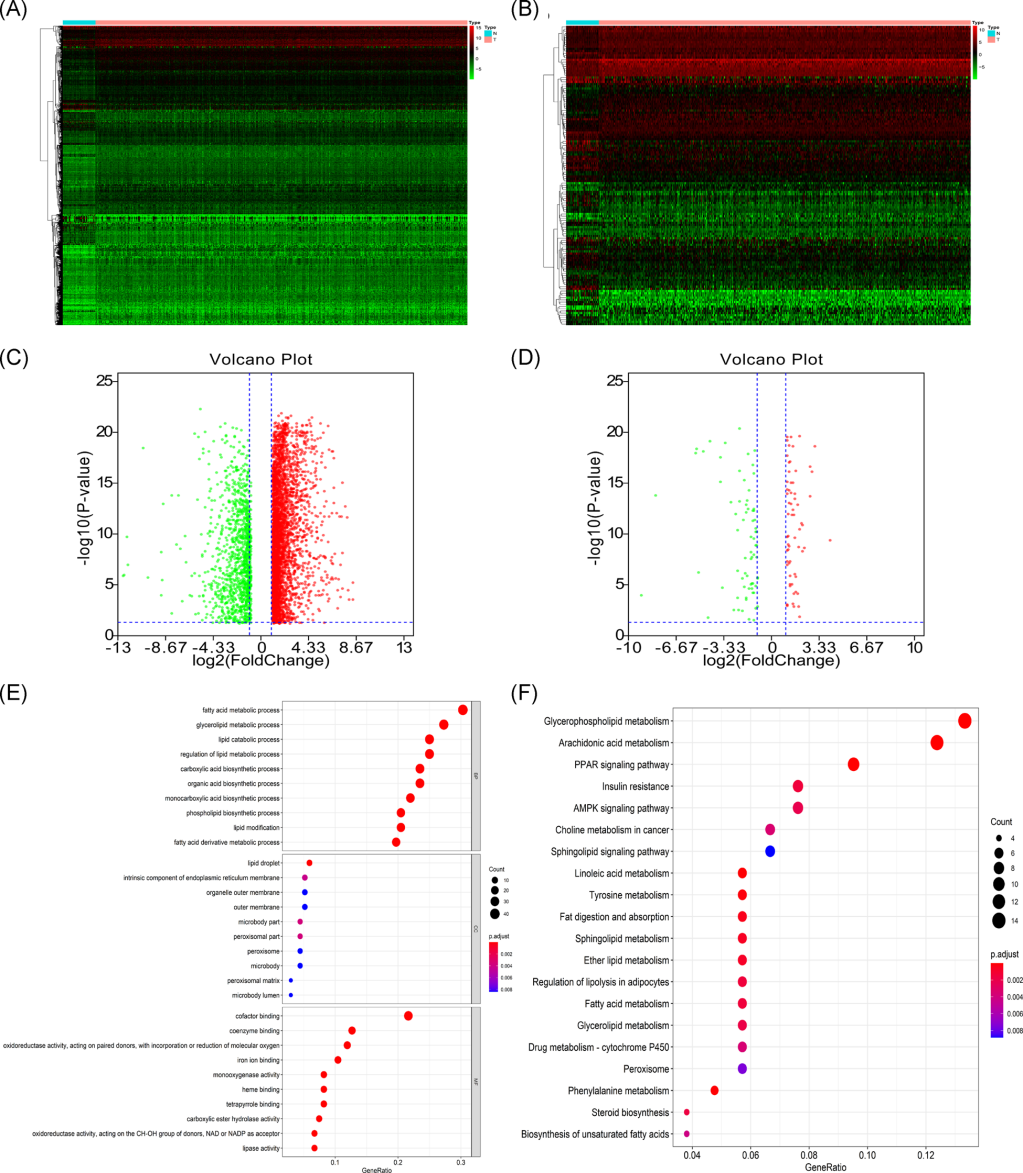
(A) Heatmap of DEGs between tumor and matched adjacent normal tissue. (B) Heatmap of lipid metabolism‐related DEGs between tumor and matched adjacent normal tissue. (C) Volcano map of DEGs. Red represent 3602 upregulated DEGs. Green represent 1181 downregulated DEGs. (D) Volcano map of lipid metabolism‐related DEGs. Red represent 64 upregulated lipid metabolism‐related DEGs. Green represent 72 downregulated lipid metabolism‐related DEGs. (E) GO term enrichment analysis of lipid metabolism‐related DEGs. (F) KEGG pathway enrichment analysis of lipid metabolism‐related DEGs. DEGs, differentially expressed genes; GO, Gene Ontology; KEGG, Kyoto Encyclopedia of Genes and Genomes

There were 4783 HNSCC DEGs, of which 136 are lipid metabolism‐related genes. A total of 3602 DEGs were highly expressed and 1181 lowly expressed (Figure 1A–C). 64 differentially expressed lipid metabolism‐related genes were upregulated and 72 downregulated (Figure 1D). GO term analysis of these lipid metabolism‐related DEGs revealed that “fatty acid metabolic process,” “lipid droplet,” and “cofactor binding” were commonest in biological progress, cellular components, and molecular functions (MF), respectively (Figure 1E). The KEGG pathway analysis confirmed that glycerol phospholipid metabolism was the most abundant pathway (Figure 1F).

### 3.3 Mutation of prognosis‐associated lipid metabolism‐related DEGs

**Figure 2:**
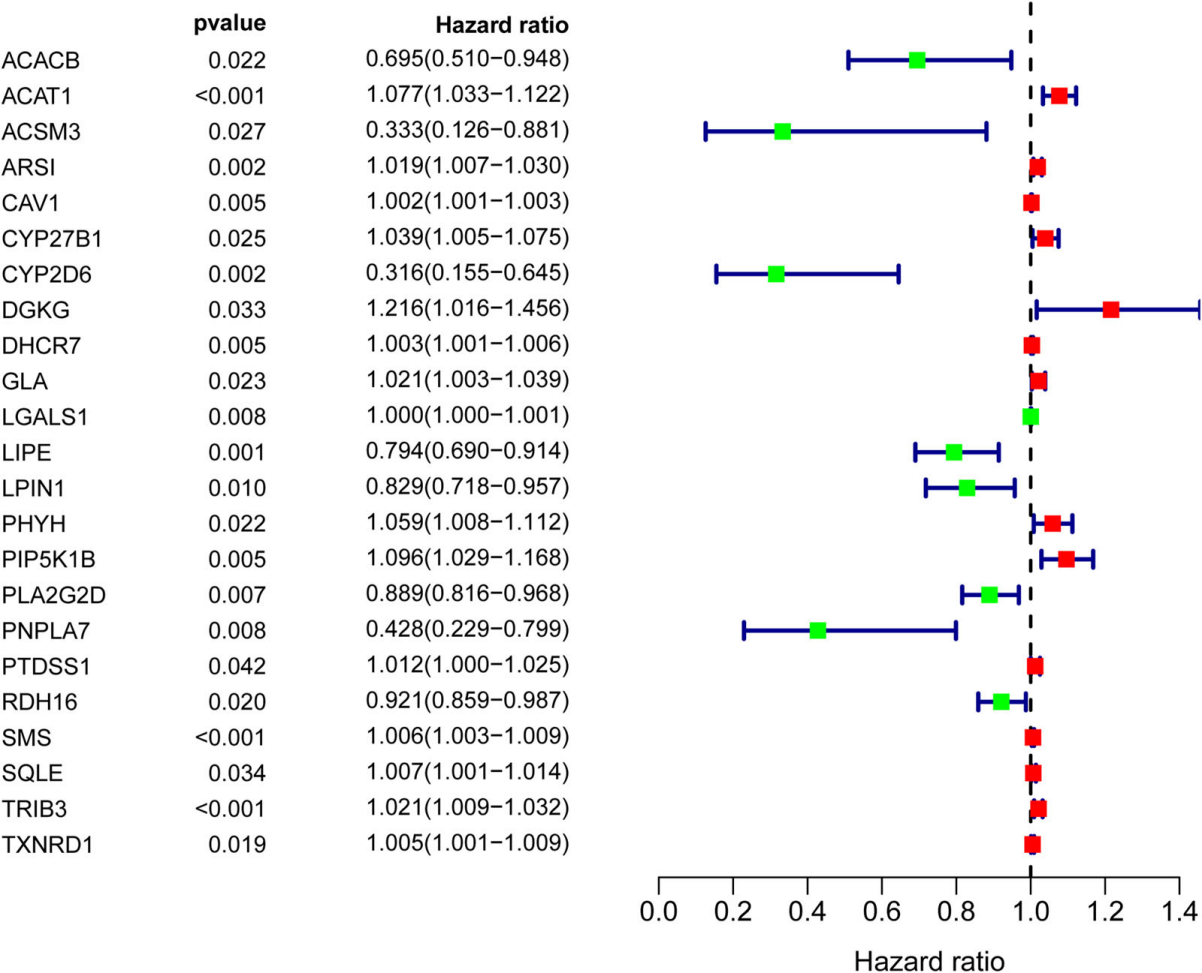
Prognosis‐associated lipid metabolism‐related differentially expressed genes

**Figure 3:**
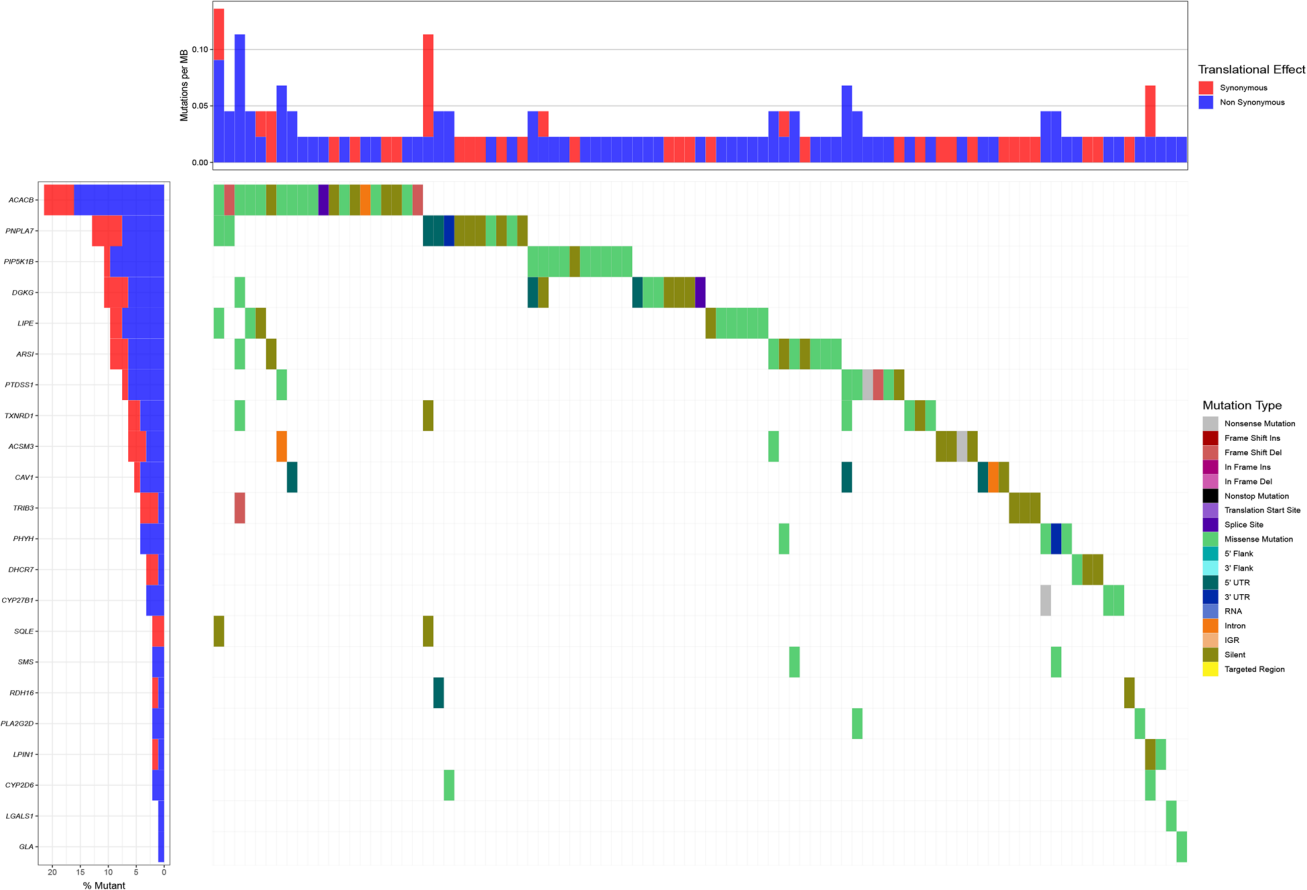
Mutation analysis of prognosis‐associated lipid metabolism‐related differentially expressed genes

Univariate analysis identified 23 prognosis‐related lipid metabolism genes (Table 1). Forest map analysis found that 14 prognosis‐related lipid metabolism genes were high‐risk genes (Figure 2). Mutation analysis revealed that many prognosis‐related lipid metabolism genes had missense mutations (Figure 3).

**Table 1:** Characteristics of prognostic‐associated lipid metabolism‐related DEGs in HNSCC

Gene symbol	logFC	FDR	HR	*p*‐value
ACACB	−1.91283	2.06E‐08	0.695329	.021644
ACAT1	−1.07091	1.03E‐09	1.076735	.000427
ACSM3	−1.81226	6.40E‐13	0.332704	.026772
ARSI	1.845219	1.59E‐09	1.018558	.00167
CAV1	2.022183	7.76E‐12	1.001701	.005342
CYP27B1	2.655062	2.14E‐17	1.039416	.025136
CYP2D6	1.038407	0.000924	0.31642	.001544
DGKG	1.650183	4.49E‐05	1.216316	.033068
DHCR7	1.201921	4.21E‐08	1.003386	.00505
GLA	1.011635	5.67E‐16	1.020917	.023121
LGALS1	1.756956	1.18E‐13	1.000487	.008192
LIPE	−1.07213	1.53E‐08	0.794247	.00126
LPIN1	−1.35084	4.47E‐13	0.829065	.010292
PHYH	−1.81438	0.00023	1.059175	.021776
PIP5K1B	−1.17052	0.001633	1.096121	.004707
PLA2G2D	1.915179	0.010662	0.888622	.006509
PNPLA7	−1.7196	2.77E‐13	0.42771	.007787
PTDSS1	1.049457	1.72E‐18	1.012456	.042013
RDH16	2.101086	1.06E‐11	0.92112	.020293
SMS	1.264379	6.68E‐18	1.006017	.000451
SQLE	1.050198	1.72E‐10	1.007276	.033865
TRIB3	1.478643	1.10E‐12	1.020547	.000329
TXNRD1	1.252268	7.86E‐06	1.004885	.018553

Abbreviations: DEGs, differentially expressed genes; FC, fold change; FDR, false discovery rate; HNSCC, Head and neck squamous cell carcinoma; HR, hazard ratio.

### 3.4 Transcriptional regulation of prognosis‐associated lipid metabolism‐related genes

**Figure 4:**
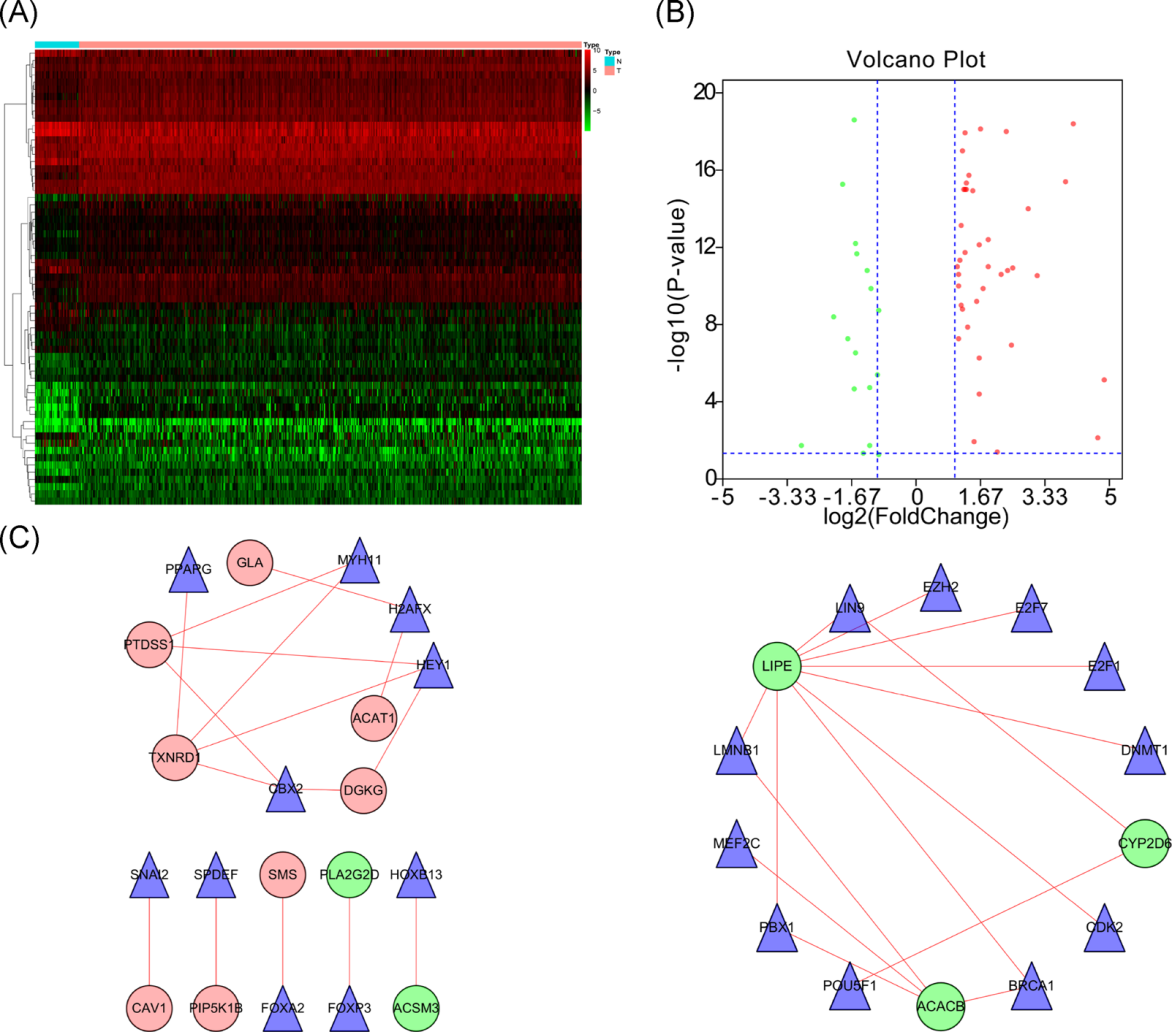
Transcriptional regulation of lipid metabolism‐related genes. (A) Heatmap of differentially expressed TFs between tumor and matched adjacent normal tissue. (B) Forty‐six upregulated DEGs (red) and 17 downregulated DEGs (green). (C) TFs and prognosis related lipid metabolism genes regulatory network. DEGs, differentially expressed genes; TFs, transcription factors

A total of 63 DEGs were TFs (Figure 4A), of which 46 were highly expressed and 17 lowly expressed (Figure 4B). Transcription regulation results showed that eigth high‐risk lipid metabolism‐related genes (GLA, ACAT1, DGKG, TXNRD1, PTDSS1, SMS, PIP5K1B, and CAV1) and eight TFs (PPAPG, MYH11, H2AFX, HEY1, CBX2, SNAI2, SPDEF, and FOXA2) were strongly associated with poor HNSCC prognosis. Moreover, five low‐risk lipid metabolism‐related genes, including LIPE, CYP2D6, ACACB, FLA2G2D, and ACSM3, were regulated by 13 TFs (FOXP3, HOXB13, LIN9, EZH2, E2F7, E2F1, DNMT1, CDK2, BRCA1, POU5F1, FBX1, MEF2C, and LMNB1; Figure 4C).

### 3.5 Establishment of a lipid metabolism‐related genes model

**Figure 5:**
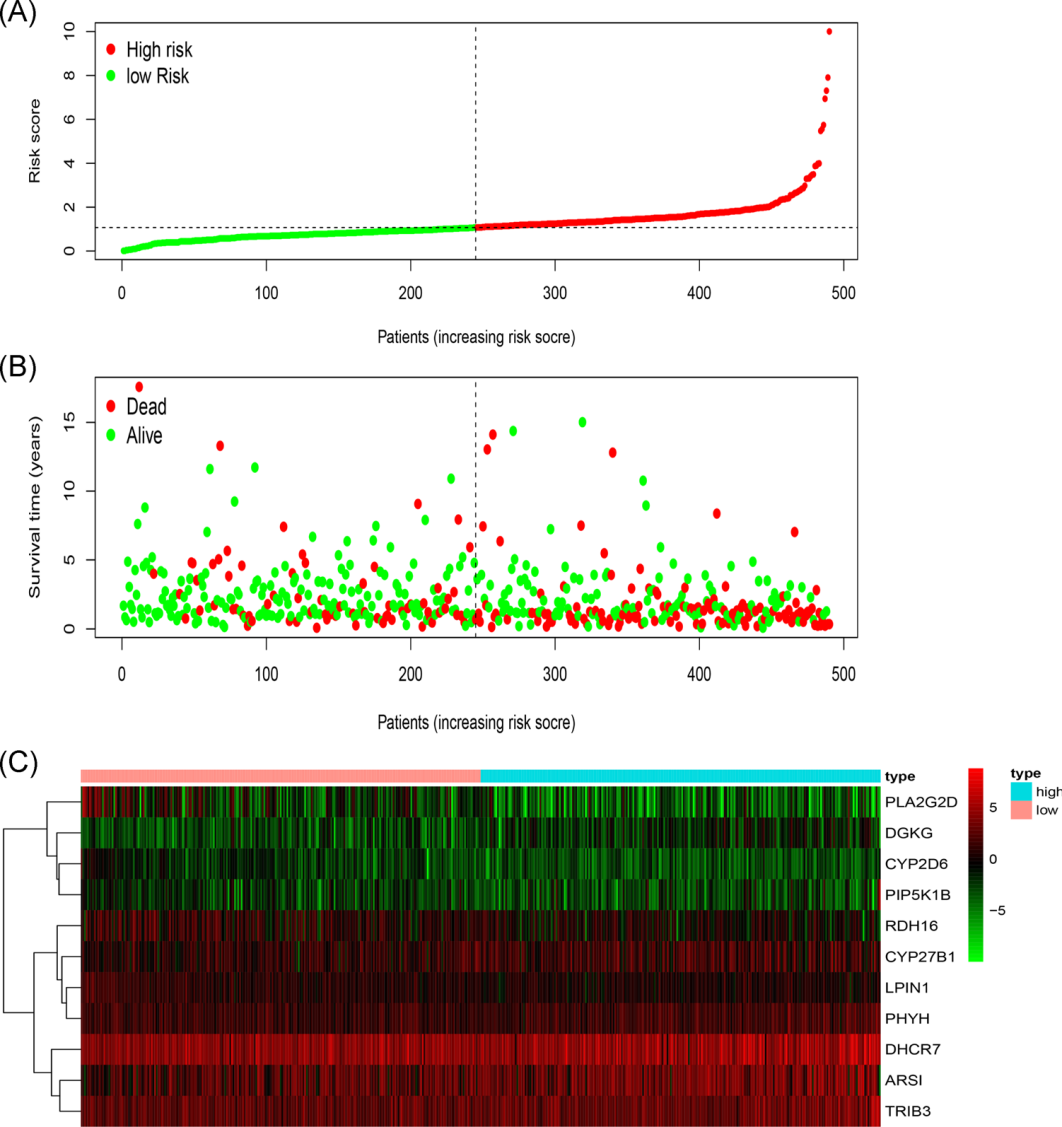
Establishment of a prognosis‐associated lipid metabolism‐related genes model. (A) Rank and distribution of prognostic index. (B) Survival status of patients in two groups. (C) Heatmap of the signature genes

**Figure 6:**
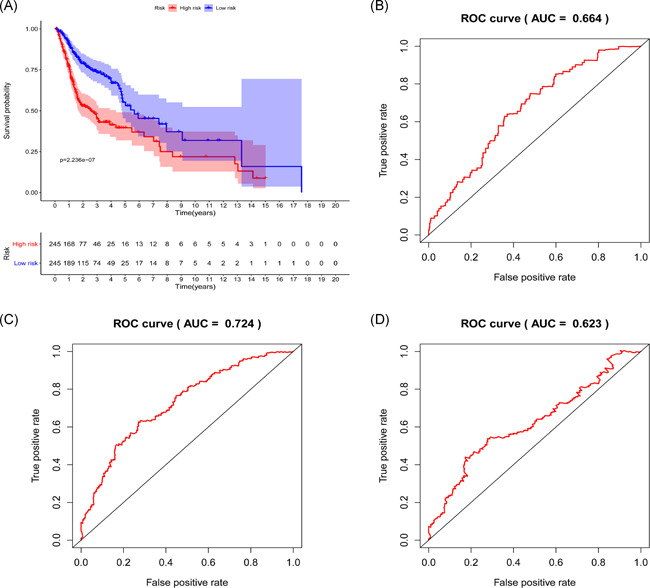
Survival prediction and model validation. (A) Prediction of outcome of stratified patients. (B–D) 1‐, 3‐, and 5‐year AUCs were 0.664, 0.724, and 0.623, respectively. AUC, area under the curve; ROC, receiver operating characteristic

**Figure 7:**
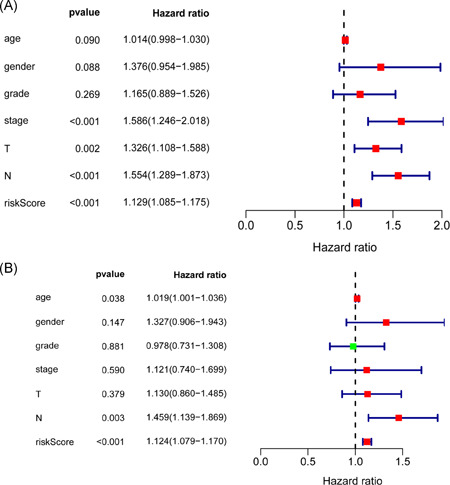
Risk score independent prognostic analysis. (A) Univariate regression analysis of HNSCC. (B) Multiple regression analysis of HNSCC. HNSCC, head and neck squamous cell carcinoma

**Figure 8:**
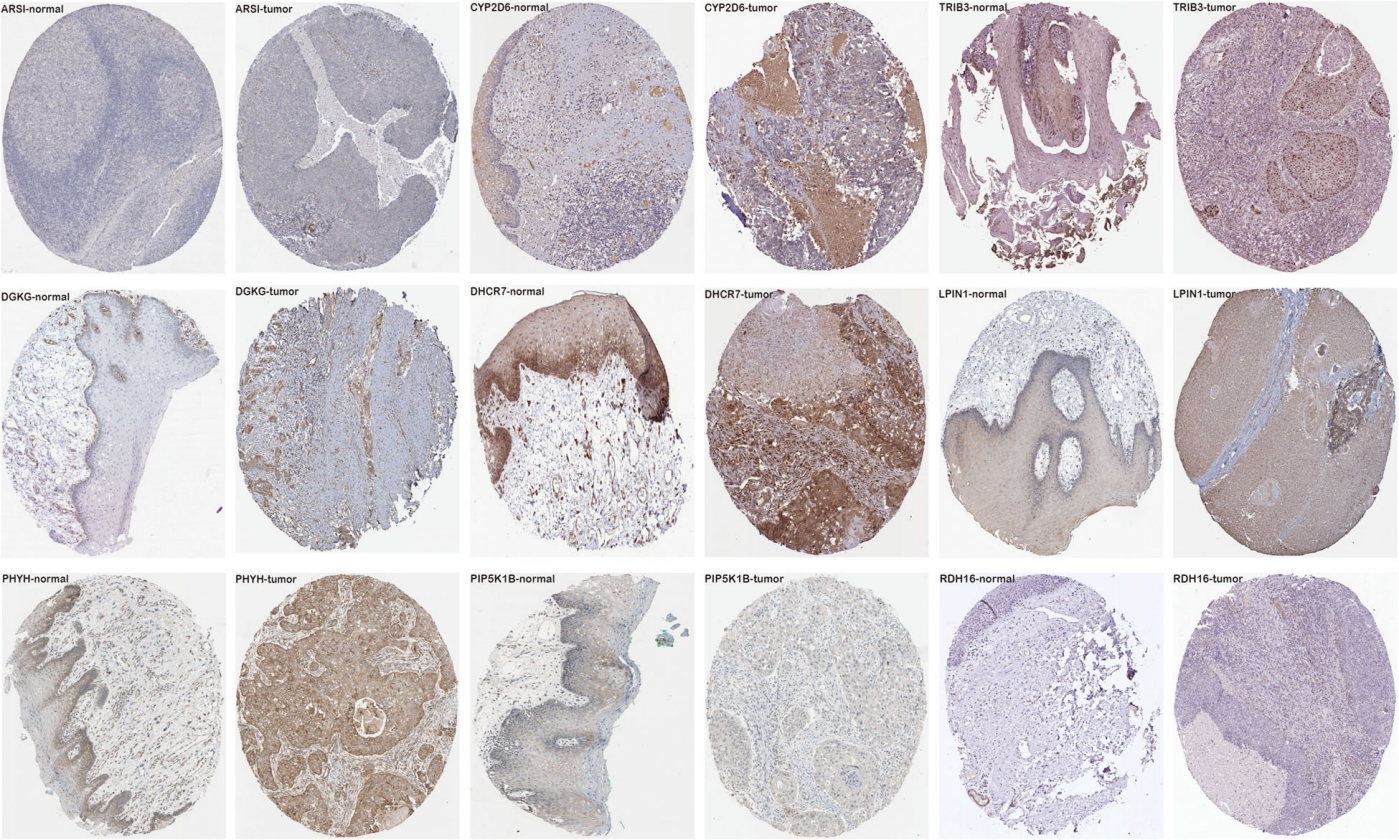
Immunohistochemistry of the gene set on HPA. Representative images showing the expression of each gene in HNSCC tissues versus normal oral cavity mucosal tissues. HNSCC, head and neck squamous cell carcinoma; HPA, The Human Protein Atlas

The PI of each patient was calculated using a Cox regression model as follows: [Expression level of ARSI × (0.0174)] + [Expression level of CYP27B1 × (0.0351)] + [Expression level of CYP2D6 × (−0.7896)] + [Expression level of DGKG × (0.2370)] + [Expression level of DHCR7 × (0.0038)] + [Expression level of LPIN1 × (−0.1588)] + [Expression level of PHYH × (0.0667)] + [Expression level of PIP5K1B × (0.0959)] + [Expression level of PLA2G2D × (−0.0737) + [Expression level of RDH16 × (−0.1001)] + [Expression level of TRIB3 × (0.0138)]. Next, patients were divided into high‐ and low‐risk group based on median PI and a risk curve constructed (Figure 5A–C). Figure 6A shows that low‐risk patients had higher survival rates relative to high‐risk patients. The PI based on lipid metabolism‐related genes can distinguish patients with HNSCC based on potential discrete clinical outcomes. Thus, we established that PI effectively and accurately stratifies patients with HNSCC. Area under the curve values of the 1‐, 3‐, and 5‐year were 0.664, 0.724, and 0.623, respectively (Figure 6B–D), indicating a high prognostic value. Univariate and multivariate analyses were used to estimate the value of the acquired PI (Figure 7). Univariate risk analysis identified clinical stage, N stage and PI, as independent prognosis predictors. However, comprehensive analysis of all clinical information indicated that only PI was an independent predictor (*p* < .001). HPA database IHC data validated the risk model, except for CYP27B1 and PLA2G2D (Figure 8).

### 3.6 Correlation analysis of clinical and immune cell infiltration

**Figure 9:**
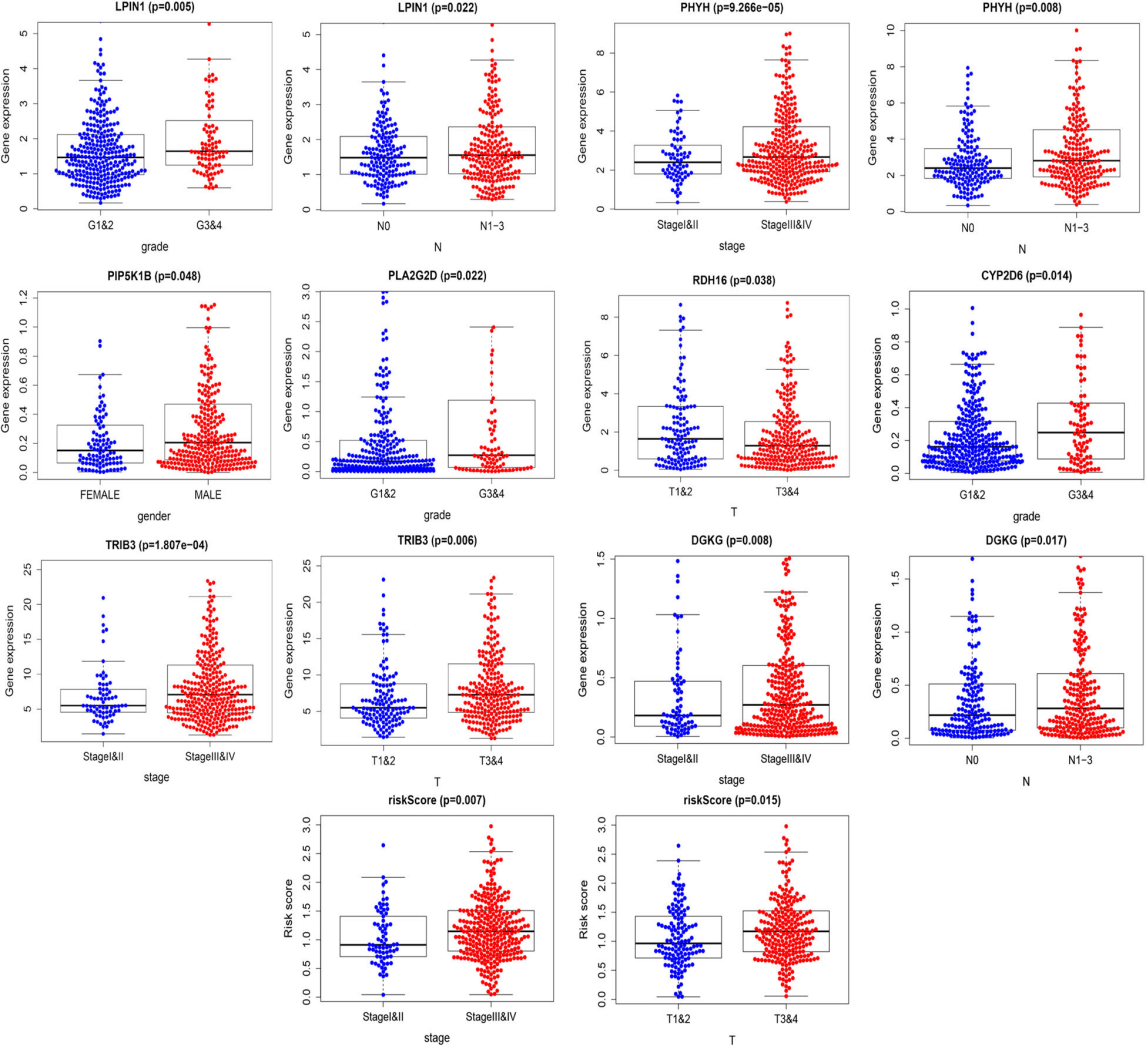
Relationship between risk genes and head and neck squamous cell carcinoma clinical features

**Figure 10:**
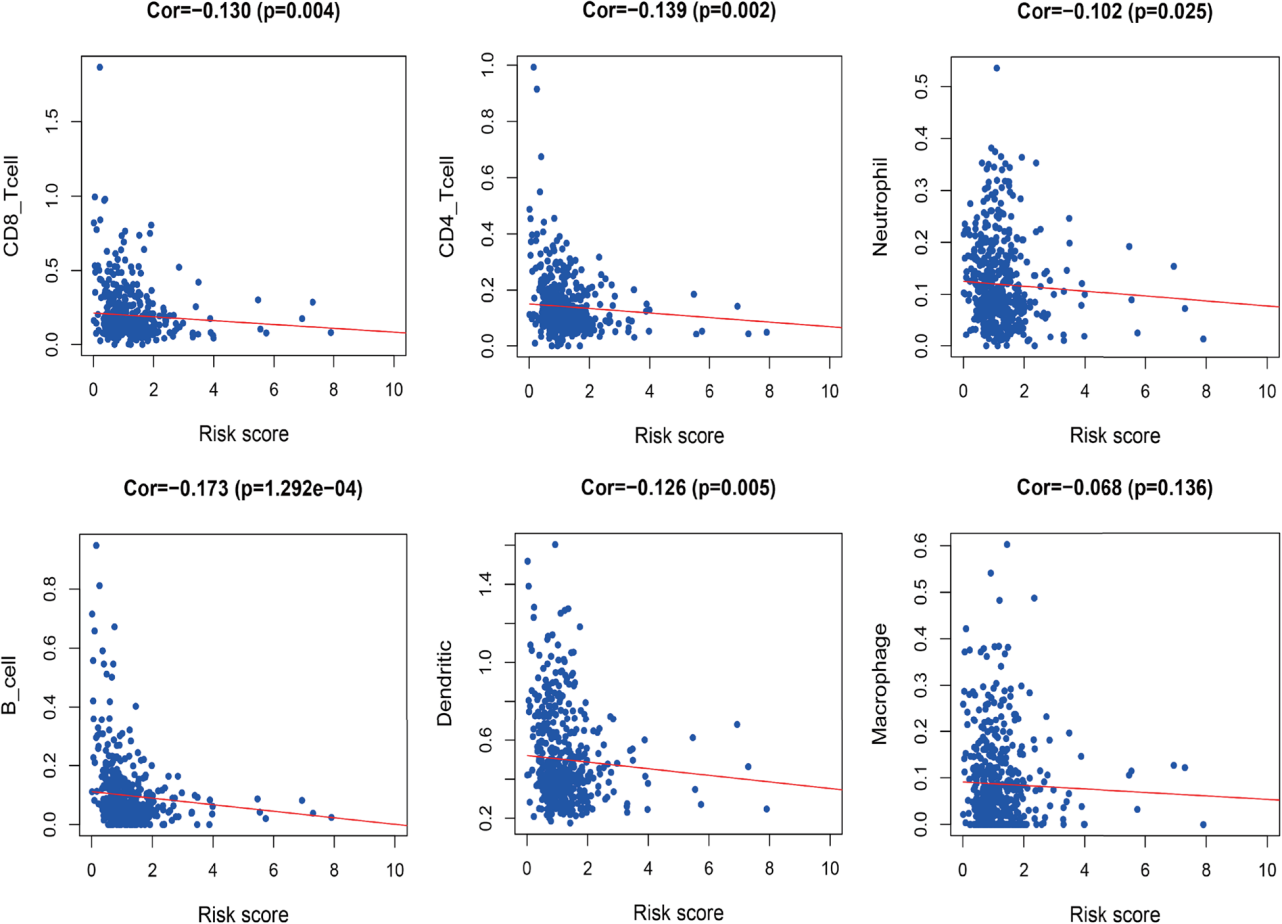
Relationships between risk score and six immune cell type in head and neck squamous cell carcinoma

Multivariate Cox regression analysis of the relationship between lipid metabolism‐related genes and clinical characteristics like age, sex, pathological stage, clinical stage, T stage, and N stage (Table 2) revealed statistically significant association with different genes (Figure 9). Analysis of the relationship between risk genes and clinical features revealed that CYP2D6, LPIN1, and PLA2G2D were highly expressed in G3&4 relative to G1&2 (*p* = .014, *p* = .005, and *p* = .022). PIP5K1B was highly expressed in females (*p* = .048). DGKG, PHYH, TRIB3, and risk score positively correlated with clinical stage (*p* = .008, *p* < .001, *p* < .001, and *p* = .007). Risk score and TRIB3 were positively correlated with T stage (*p* = .006 and *p* = .015), while RDH16 negatively correlated with T stage (*p* = .038). DGKG, LPIN1, and PHYH positively correlated with N stage (*p* = .017, *p* = .022, and *p* = .008). Examination of the relationship between PI and immune cell infiltration to determine if these risk genes precisely reflect HNSCC immune environment revealed that lipid metabolism‐related genes PI negatively correlated with CD4^+^ T‐cells, CD8^+^ T‐cells, neutrophils cell, B‐cells, and dendritic cells (*p* = .002, *p* = .004, *p* = .025, *p* < .001, and *p* = .005; Figure 10).

**Table 2:** Relationship between risk genes and HNSCC clinical features

	Age	Gender	Grade	Stage	T	N
Gene symbol	(≤ 60/>60)	(Male/Female)	(1&2/3&4)	(I&II/III&IV)	(1&2/3&4)	(0/1–3)
	*t*(*p*)	*t*(*p*)	*t*(*p*)	*t*(*p*)	*t*(*p*)	*t*(*p*)
ARSI	0.233	−0.028	1.051	−0.324	−0.528	−0.19
	(0.816)	(0.978)	(0.295)	(0.747)	(0.598)	(0.849)
CYP27B1	−0.749	1.221	−0.391	1.401	−0.293	0.617
	(0.455)	(0.224)	(0.696)	(0.164)	(0.770)	(0.537)
CYP2D6	1.218	−1.074	−**2.485**	−1.346	0.643	−1.408
	(0.224)	(0.284)	**(0.014)**	(0.181)	(0.521)	(0.160)
DGKG	−0.709	0.058	−0.741	−**2.69**	−1.767	−**2.407**
	(0.479)	(0.954)	(0.460)	**(0.008)**	(0.078)	**(0.017)**
DHCR7	1.733	−0.498	−0.047	−0.69	−1.78	−1.563
	(0.084)	(0.619)	(0.963)	(0.492)	(0.076)	(0.119)
LPIN1	0.665	−0.672	−**2.9**	−0.638	−0.912	−**2.295**
	(0.507)	(0.502)	**(0.005)**	(0.524)	(0.362)	**(0.022)**
PHYH	−0.782	−0.803	−1.942	−**3.975**	0.126	−**2.678**
	(0.435)	(0.423)	(0.055)	**(9.266e‐05)**	(0.900)	**(0.008)**
PIP5K1B	0.838	−**1.981**	−0.996	−1.95	−1.399	−1.443
	(0.403)	**(0.048)**	(0.322)	(0.052)	(0.163)	(0.150)
PLA2G2D	1.287	−1.377	−**2.324**	0.787	1.793	−0.312
	(0.199)	(0.169)	**(0.022)**	(0.434)	(0.075)	(0.756)
RDH16	0.117	−0.512	0.101	1.06	**2.093**	0.219
	(0.907)	(0.609)	(0.920)	(0.292)	**(0.038)**	(0.827)
TRIB3	−0.351	−1.077	−0.329	−**3.793**	−**2.759**	−0.632
	(0.726)	(0.283)	(0.743)	**(1.807e‐04)**	**(0.006)**	(0.528)
Risk score	0.912	−0.316	−0.881	−**2.701**	−**2.438**	−1.537
	(0.363)	(0.752)	(0.381)	**(0.007)**	**(0.015)**	(0.126)

*Note*: The bold values represent the significant *t* and *P* value of the comparison between the two groups. *t, t* value of Student's *t* test; *p: p*‐value of Student's *t* test.

Abbreviation: HNSCC, head and neck squamous cell carcinoma.

## 4 DISCUSSION

HNSCC is characterized by a low cure rate and easy recurrence. Multiple studies have shown that aberrant lipid metabolism influences HNSCC tumorigenesis. In HNSCC models, cetuximab resistance has been linked to lipid metabolism alterations.^15^ Reduced expression of metabolism‐related genes may improve survival of HPV‐positive patients with head and neck cancer (HNC).^16^ FADS1 has emerged as mediator of lipid metabolism gene and drives laryngeal squamous cell carcinoma progression by activating AKT/mTOR signaling pathway.^17^ In oral squamous cell carcinoma (OSCC), the Kennedy signaling pathway is upregulated by cholesterol and glycerophospholipid (GPL) metabolic changes.^18^ While lipid metabolism is vital for HNC, its role is not completely understood in HNSCC.

Here, we used bioinformatics to comprehensively study lipid metabolism‐related genes in HNSCC. Our data highlight the value of these genes in anti‐HNSCC therapy. We confirmed that some lipid metabolism‐related genes are involved in HNSCC progression and constructed individualized lipid metabolism prognostic indicators to assess immune cells infiltration and survival rate. Thus, lipid metabolism‐related genes are potential tumor biomarkers.

RNA‐seq analysis of lipid metabolism‐related genes in HNSCC tumor tissue versus adjacent noncancer tissue found that lipid metabolism‐related genes are altered in HNSCC. These DEGs exhibit enrichment for fatty acid metabolic processes. Elevated fatty acid synthesis has been observed in various cancers. Multiple studies have confirmed that lipogenesis is important for tumor growth.^19^ The MFs analysis revealed cofactor binding enrichment. An active RNA editing complex is formed by APOBEC1 cytosine deaminase and cofactor A1CF. This complex acts on APOB RNA to regulate lipid metabolism.^20^ The KEGG pathway analysis revealed GPL metabolism as the most enriched pathway. In OSCC, GPL metabolism is the most abundant form of lipid metabolism.^18^ These data indicated the molecular mechanism underlying lipid metabolism in HNSCC.

Univariate Cox analysis revealed that 23 lipid metabolism‐related genes correlate with HNSCC prognosis. Of these, acetyl‐coA acetyltransferase 1 (ACAT1), diacylglycerol kinase gamma (DGKG), and phosphatidylinositol‐4‐phosphate 5‐kinase type 1 beta (PIP5K1B) genes had higher hazard ratios. These genes have been reported to influence carcinoma tumorigenesis, proliferation, and migration.^21^, ^22^, ^23^, ^24^, ^25^, ^26^, ^27^, ^28^ It has been reported that missense mutations are common in HNSCC cell lines.^29^ Fatty acid and cholesterol metabolism are regulated by TFs.^1^ Thus, we developed a regulatory network of TFs‐lipid metabolism genes and identified thioredoxin reductase‐1, phosphatidylserine synthase 1, and DGKG as the key regulatory TFs genes. These findings are consistent with past studies,^30^, ^31^, ^32^, ^33^, ^34^, ^35^ indicating the reliability of our study.

Cox regression analysis revealed 11 lipid metabolism‐related genes associated with HNSCC prognosis. ROC indicated that our results are highly accurate. Lipid metabolism‐related genes PI had a high prognosis value and were related to age, pathological tumor stage, TN stage, and gender. It is reported that CYP27B1, CYP2D6, and TRIB3 are directly or indirectly involved in HNSCC development and progression.^36^, ^37^, ^38^, ^39^ DGKG, DHCR7, LPIN1, PIP5K1B, PLA2G2D, and RDH16 are involved in the development of other tumors.^26^, ^28^, ^40^, ^41^, ^42^, ^43^ However, ARSI and PHYH have not been previously studied in cancers. In addition, the gene set was validated using HPA data on HNSCC and other tumors. Further analysis of the relationship between these genes will offer theoretical basis for HNSCC treatment.

27‐hydroxyl cholesterol indirectly affects breast cancer metastasis by increasing the number of metastasizing immune cells and suppressing CD8^+^ T‐cells.^44^ Increased TIME cholesterol may cause CD8^+^ T‐cell exhaustion.^45^ Together, these findings indicate that abnormal lipid metabolism may affect the number and function of tumor immune cells. Thus, we designed a simple protocol for monitoring immune status in patients with HNSCC based on lipid metabolism‐associated genes. The results indicated that CD4^+^ T‐cells, CD8^+^ T‐cells, neutrophils, B‐cells, and dendritic cells had a higher degree of infiltration in low‐risk patients with HNSCC. Consistent with past studies,^46^, ^47^, ^48^, ^49^ we found that CD4^+^ T‐cells and CD8^+^ T‐cells were necessary for antitumor immunity and improved prognosis outcome. Neutrophils, B‐cells, and dendritic cells were comprised of different subpopulations with pro‐ and antitumor function. Here, we found that these three immune cell types have antitumor roles in HNSCC.^50^, ^51^, ^52^, ^53^, ^54^, ^55^, ^56^, ^57^, ^58^ In addition, tumor associated macrophages may be polarized into M1‐like macrophages with anticancer activity, or M2 macrophages with pro‐cancer roles.^59^ We found that macrophages (predominantly M1) have a higher degree of infiltration in low‐risk HNSCC. Our findings validate and enhance the recognition of immune cells functions in HNSCC.

In summary, we conducted a comprehensive analysis of HNSCC mutations, the regulatory mechanisms of lipid metabolism‐related genes, the relationship between these genes and clinical prognosis and immune infiltration, and determined the genes' prognostic value. The risk score of lipid metabolism‐related genes may reflect the prognosis and immune status of patients. However, the prognostic value of lipid metabolism‐related genes in HNSCC needs further experimental verification.

## CONFLICTS OF INTEREST

All the authors declare that there are no conflicts of interest.

## AUTHOR CONTRIBUTIONS

*Conceptualization, software, and methodology*: Ying Xiong and Yu Si. *Validation, investigation, and data curation*: Ying Xiong, Yu Si, and YiSi Feng. *Writing—original draft preparation*: Ying Xiong, Yu Si, Shipei Zhuo, and Bozhen Cui. *Supervision and funding acquisition*: Ying Xiong, Yu Si, and Zhigang Zhang. All authors have read and agreed to the published version of the manuscript.

## Data Availability

The authors confirm that the data supporting the findings of this study are available within the article.
